# Role of *Candida albicans-*Secreted Aspartyl Proteinases (Saps) in Severe Early Childhood Caries

**DOI:** 10.3390/ijms150610766

**Published:** 2014-06-13

**Authors:** Wenqing Li, Dongsheng Yu, Shuo Gao, Jiacheng Lin, Zhuoyu Chen, Wei Zhao

**Affiliations:** Guanghua School of Stomatology, Guangdong Provincial Key Laboratory of Stomatology, Sun Yat-sen University, Guangzhou 510055, China; E-Mails: wendysums@hotmail.com (W.L.); yudsh@mail.sysu.edu.cn (D.Y.); gshuo@mail3.sysu.edu.cn (S.G.); linjiach@mail.sysu.edu.cn (J.L.); chzhuoy@mail2.sysu.edu.cn (Z.C.)

**Keywords:** severe early childhood caries, *Candida albicans*, Saps, protease activity, genotype

## Abstract

*Candida albicans* is strongly associated with severe early childhood caries (S-ECC). However, the roles of secreted aspartyl proteinases (Saps), an important virulence factor of *C. albicans*, in the progress of S-ECC are not clear. In our study, the Saps activities were evaluated by the yeast nitrogen base–bovine serum albumi (YNB–BSA) agar plate method and by the MTT method with bovine serum albumin (BSA) as the substrate. Genotypes of *C. albicans* and gene expression of *Sap*1–5 were evaluated. The relationships of Saps activities and genotypes with S-ECC were analyzed. The results showed that enzyme activities of Saps in the S-ECC group were significantly higher than those in the caries free (CF) group (*p* < 0.05). Genotypes A, B and C were detected in the S-ECC group, and genotypes A and C were detected in the CF group. In the genotype A group, Saps activity in the S-ECC group was significantly different from that in the CF group (*p* < 0.05). The gene expression level of *Sap1* in the S-ECC group was significantly higher than that in the CF group (*p* = 0.001), while *Sap4* expression was significantly lower than that in the CF group (*p* = 0.029). It can be concluded that *Sap*1–5 are the predominant proteinase genes expressed in *C. albicans* from dental biofilm and *Sap1* may play an important role in the development of S-ECC.

## 1. Introduction

Severe early childhood caries (S-ECC) is defined in 3- to 5-year-old children as one or more cavitated teeth, teeth missing due to caries or filled smooth surfaces in primary maxillary anterior teeth, or a decayed/missing/filled tooth score of ≥4 (age 3 years), ≥5 (age 4 years) or ≥6 (age 5 years) [1]. It is a particularly aggressive form of dental caries affecting the primary teeth of young children and has become a serious public health problem. It is well-known that the mutans streptococci, including *Streptococcus mutans* and *Streptococcus sobrinus*, are the major pathogens of S-ECC [2,3,4]. Apart from this, many researchers have focused on other species involved in the microbial etiology of S-ECC [5,6]. Analysis of epidemiological data has shown that *Candida albicans* can be detected in saliva, dental plaque and caries lesions. The frequency of *C. albicans* in samples obtained from those in the ECC group was higher compared with that from the caries-free group [4,7,8,9]. Therefore, it is suggested that there is a significant association between the presence of *C. albicans* and early childhood caries.

The secreted aspartyl proteinases (Saps) are among the most important virulence factors of *C. albicans*, and are related to the adhesion of *C. albicans* to tooth surfaces and the degradation of extracellular matrix and proteins. The SAPS proteins, encoded by a family of 10 *Sap* genes [10,11], can be classified into several distinct groups according to sequence homology. The different members of the Sap family might be differentially expressed depending on environment and host conditions. For example, in the study by Naglik *et al*. [12], saliva from patients with oral *C. albicans* infection and those with asymptomatic Candida carriage was collected, and the results suggested that *Sap2* and *Sap4* to *Sap*6 were the predominant proteinase genes expressed in the oral cavity of patients with oral candidiasis and those who were Candida carriers. *Sap1* and *Sap3* transcripts were observed only in affected patients. In another study, carried out in a reconstituted human epithelium (RHE) model, the results showed that expression of *Sap1* and *Sap3* was higher than that of *Sap2* and *Sap6* [13]. Until now, such research has focused mainly on the transcript levels of SAPS from oral candidiasis, vaginal candidiasis, oropharyngeal candidiasis and gastrointestinal candidiasis [12,14,15,16,17]. However, the enzyme activity and gene expression of *Saps* in samples obtained from dental biofilm and their relationship with S-ECC are still unknown.

It is hypothesized that, in different genotypes of *C. albicans*, the enzyme activity and gene expression of *Saps* associated with the development of S-ECC are variable.

In this study, strains and genotypes of *C. albicans* from dental biofilm of children with S-ECC were isolated and identified. We detected the enzyme activity of Saps by both the yeast nitrogen base-bovine serum albumi YNB–BSA agar plate method and by the MTT method, with BSA as the substrate. Gene expression of *Sap*1–5 in genotypes of *C. albicans* was evaluated by reverse transcription polymerase chain reaction (RT-PCR).

## 2. Results and Discussion

### 2.1. The Enzyme Activity of Saps Determined by the Yeast Nitrogen Base–Bovine Serum Albumi (YNB–BSA) Agar Plate Method

**Figure 1:**
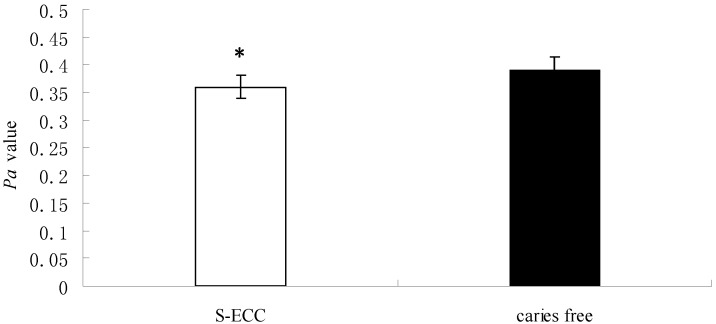
The comparison of *Pa* value and Saps activity of *Candida albicans* strains isolated from severe early childhood caries (S-ECC) and caries-free children. White bars and black bars represent the means (with standard deviations) of *Pa* values (the ratio of the diameter of the colony to the total diameter of the colony plus the zone of precipitation), which represents Saps activity of *C. albicans* from S-ECC and caries free (CF) children. * denotes statistical significance (*p* < 0.05).

All 40 strains of *C. albicans* isolated from S-ECC and caries-free children grew well on the YNB–BSA agar plate. The yeast colony was round, white and smooth-surfaced. Moreover, a halo around the colony could be seen in all *Candida* strains from both the S-ECC and the caries-free groups. According to Price *et al.* [18], enzymatic activity was determined by the ratio of the diameter of the colony to the total diameter of the colony plus the zone of precipitation (Proteolytic activity, Pa). In our study, the colony diameter in the S-ECC group was 5.63–8.00 mm, and there was no significant difference compared with that in the caries-free group, whose colony diameter was 6.42–7.42 mm (Table 1). However, the *Pa* value in the S-ECC group was significantly lower than that in the caries free (CF) group (*p* < 0.05), which means that the enzyme activity of Saps in *C. albicans* isolated from S-ECC children was higher than that from CF children (Figure 1).

**Table 1:** Saps activity determined by the yeast nitrogen base–bovine serum albumin (YNB–BSA) agar plate method.

Groups	*n*	Diameter of Colony (mm)	Diameter of Colony plus Precipitation Zone (mm)	*Pa*	*p*
S-ECC	26	7.13 ± 0.49	19.76 ± 2.63	0.36 ± 0.03	0.031
Caries-free	14	6.78 ± 0.32	17.52 ± 1.65	0.39 ± 0.05	

### 2.2. The Enzyme Activity of Saps Determined by the Bovine Serum Albumin–MTT (BSA–MTT) Method

**Figure 2:**
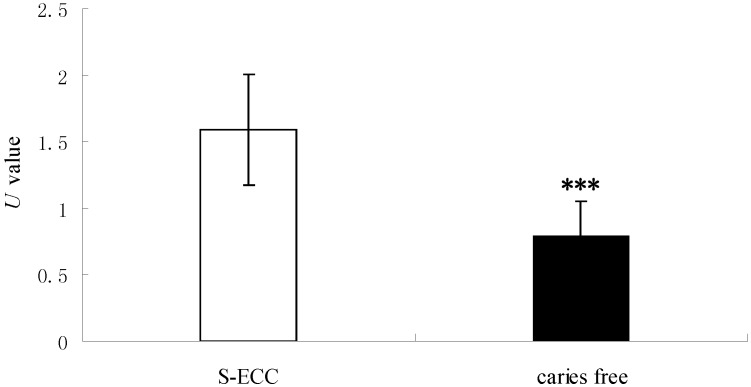
The comparison of Saps activity of *C. albicans* strains isolated from S-ECC and caries-free children as determined by the MTT method. White bars and black bars represent the means (with standard deviations) of *U* values (OD_280_/OD_490_), which represent Saps activity of *C. albicans* from S-ECC and CF children. *** denotes statistical significance (*p* < 0.001).

First, live yeast was detected by the MTT method (Table 2, Figure 2). The MTT value was higher in the CF group; however, the *U* value was significantly higher in the S-ECC group (*p* < 0.001), which means that the enzyme activity of Saps in *C. albicans* isolated from S-ECC children was higher than that from CF children.

**Table 2:** Saps activity determined by the bovine serum albumin–MTT (BSA–MTT) method.

Groups	*n*	OD_280_	MTT Value	*U*	*p*
S-ECC	26	0.53 ± 0.25	0.40 ± 0.19	1.59 ± 0.92	0.001
Caries-free	14	0.42 ± 0.13	0.54 ± 0.12	0.79 ± 0.26	

### 2.3. Genotypes of Candida albicans from Severe Early Childhood Caries (S-ECC) Children

**Figure 3:**
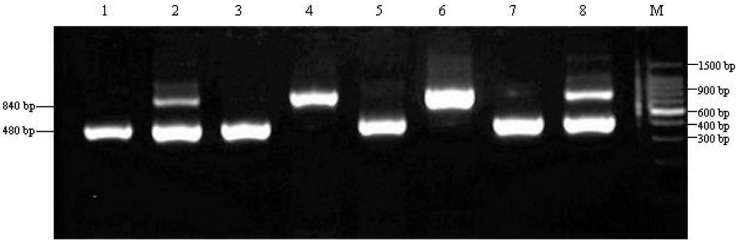
The polymerase chain reaction (PCR) characterization profiles of the genotypes of *C. albicans.* 25s rDNA. Lanes **1**, **3**, **5** and **7** were for genotype A, and the size of the PCR products was approximately 450 bp; Lanes **4** and **6** were for genotype B, and the size of the PCR products was approximately 840 bp; Lanes **2** and **8** were for genotype C, and the sizes of the two PCR products were approximately 450 and 840 bp.

To investigate whether the enzyme activity of Saps is related to the sequence of genomic DNA, we evaluated the genotypes of *C. albicans* by arbitrarily primed polymerase chain reaction (AP-PCR). According to previous studies, genotypes of *C. albicans* can be divided into five different groups, based on the length of the polymerase chain reaction (PCR) amplification product, namely, genotypes A (450 bp), B (840 bp), C (both 450 and 840 bp), D (1080 bp) and E (1400 bp) [12,13]. After all *C. albicans* colonies identified were screened, genotypes A, B and C of *C. albicans* were detected in the dental biofilms of children with S-ECC, while only genotypes A and C of *C. albicans* were detected in the dental biofilms of CF children (Table 3, Figure 3). Genotypes D and E were not detected in the oral cavities of any examined children. These results are consistent with those reported by Yang *et al.* [19].

**Table 3:** Genotypes of *C. albicans* isolated from different sampling sites in S-ECC and CF children.

Groups	*C. albicans* Isolate		Genotypes	
		A	B	C
S-ECC group	23	13 (56.5%)	7 (30.4%)	3 (13.1%)
CF group	17	8 (47.1%)	ND	9 (52.9%)

ND: Not found.

Furthermore, whether the virulence of *C. albicans* is related to genotypes remains controversial. One study found that genotype A was more prevalent among invasive isolates and that genotypes B and C were more prevalent among non-invasive isolates (*p* < 0.05) [15], while another study, conducted by Al-Karaawi *et al.*, reported that genotype A is the most predominant type in patients with oral Candida infection; however, there was no relationship with the virulence of *C. albicans* [20].

**Table 4:** Comparison of Saps activity in different genotype groups.

Genotype	Enzyme Activity	Group	*N*	*x* ± *s*	*F*	*t*	Sig.
Genotype A	*Pa*	CF	8	0.34 ± 0.03	0.426	−2.385	0.028
		S-ECC	13	0.38 ± 0.04			
	*U*	CF	8	1.33 ± 0.67	8.687	2.296	0.034
		S-ECC	13	0.82 ± 0.27			
Genotype C	*Pa*	CF	9	0.36 ± 0.04	2.029	−1.898	0.087
		S-ECC	3	0.42 ± 0.07			
	*U*	CF	9	1.73 ± 0.34	4.88	1.307	0.22
		S-ECC	3	0.68 ± 0.21			

*N* means number; *x* ± *s* denotes mean ± standard deviation; *F* denotes the result of F test; *t* denotes the result of *t* test; Sig. means significance, which is equal to *p* value.

**Figure 4:**
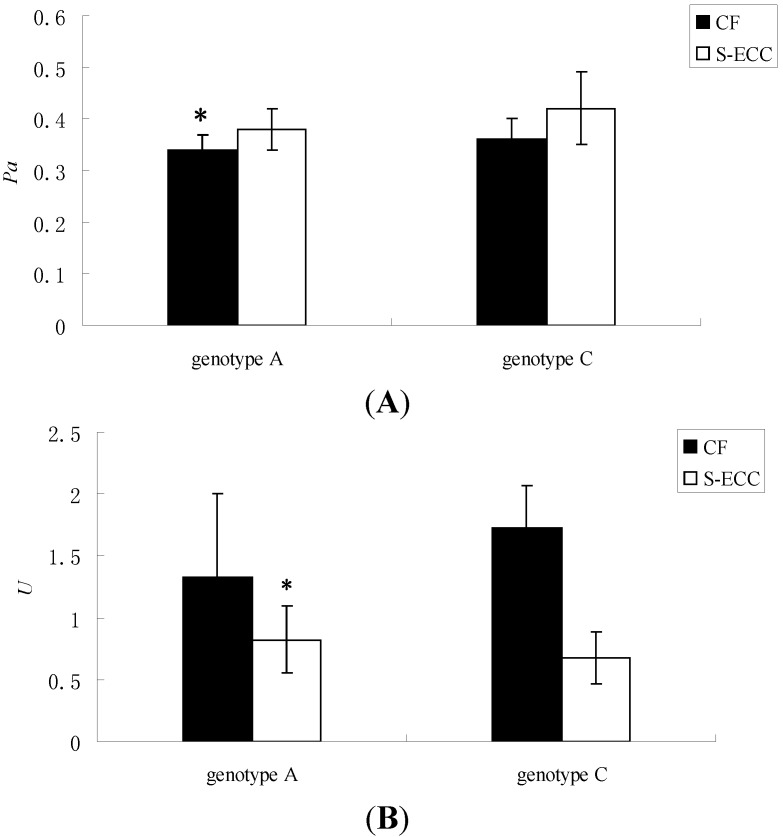
Comparison of the Saps activity in different genotype groups. White bars and black bars represent the means (with standard deviations) of *Pa* values (**A**) and *U* values (**B**), which represent the Saps activity of *C. albicans* from S-ECC and CF children. * denotes statistical significance (*p* < 0.05).

The relationship of Saps activity and the different genotypes was also analyzed. According to the results of one-way ANOVA and the Kruskal-Wallis test, in the genotype A group, the Saps activity of *C. albicans* isolated from S-ECC children was significantly different from that in CF children (*p* < 0.05) (Table 4, Figure 4). However, there was no significant difference in Saps activity in the genotype C group of *C. albicans* from S-ECC and CF children. The results supported *C. albicans* of genotype A as more aggressive in the progression of S-ECC.

### 2.4. Gene Expression of Sap1–5 in Dental Biofilm from S-ECC and CF Children

**Figure 5:**
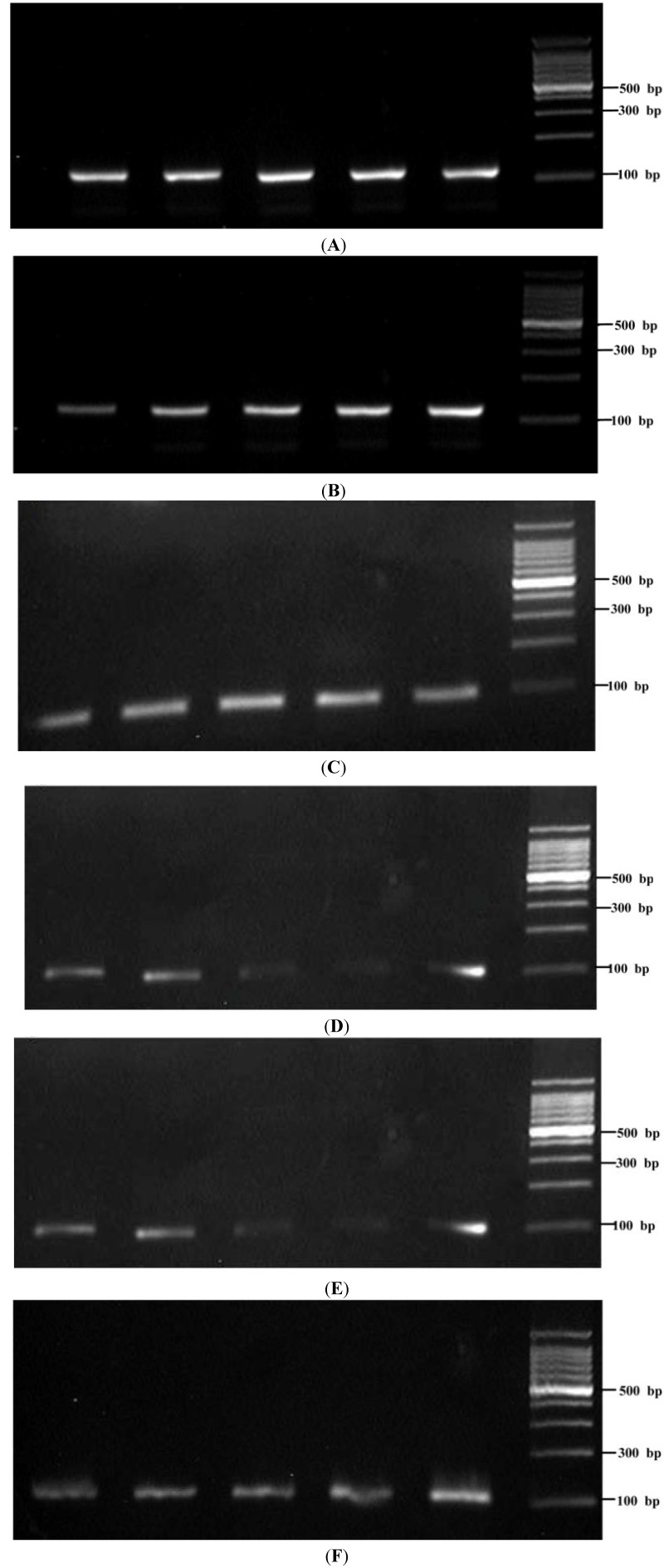
RT-PCR amplification of *Sap1* (**A**); *Sap2* (**B**); *Sap3* (**C**); *Sap4* (**D**); *Sap5* (**E**); and ACT-1 (**F**).

**Figure 6:**
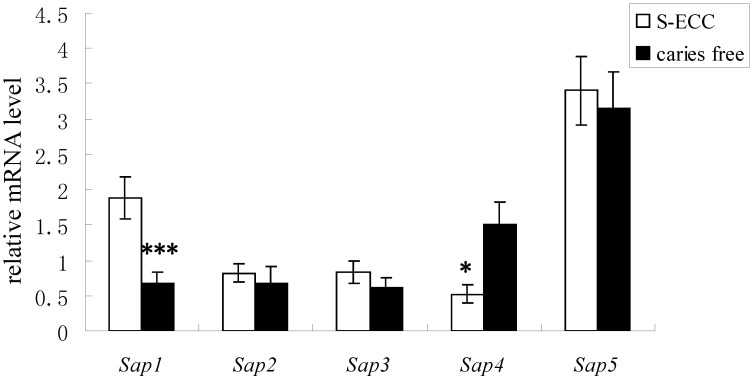
The gene expression of *Sap*1–5 in the S-ECC and caries-free groups. White bars and black bars represent the means (with standard deviations) of relative mRNA levels, which represent gene expression of Saps of *C. albicans* from S-ECC and CF children. * denotes statistical significance (*p* < 0.05), and *** denotes statistical significance (*p* < 0.001).

Until now, Saps expression has been detected in all types of *C. albicans* infections by various gene expression detection techniques. The different members of the SAPS family might be differentially expressed *in vivo* and might contribute to different *C. albicans* infections, because the pathogen inhabits a diverse number of host niches under a variety of environmental conditions [21,22]. In the study by Naglik *et al*. [14], samples were obtained from individuals with, or carrying, oral and vaginal candidiasis, and the expression of *Sap1–Sap8* was evaluated in an *in vivo* model. The results suggested that *Sap2* and *Sap5* were the most common genes expressed during oral mucosal infection. *Sap1*, *Sap3*, *Sap4*, *Sap7* and *Sap8* expression was correlated with oral mucosal infection, whereas *Sap1*, *Sap3* and *Sap6*–*Sap8* expression was correlated with vaginal disease. Another study found the expression of Saps in oropharyngeal candidiasis in a murine model [23], and the results showed sustained expression of *Sap1*–*Sap6* and *Sap9*, with *Sap5* and *Sap9* most strongly expressed throughout the course of infection. The research focused mainly on the gene expression of Saps from oral candidiasis, vaginal candidiasis, oropharyngeal candidiasis and gastrointestinal candidiasis, and the results differed considerably because of the different models involved [12,13,14,15,17,23,24]. However, the gene expression of Saps in samples obtained from dental biofilm and its relationship with S-ECC are still unclear. In our study, *Sap1–Sap5* could be detected in all of the dental biofilm samples obtained from S-ECC and CF children (Figure 5). Results showed that the highest gene expression levels were found for *Sap1* and *Sap5* in the S-ECC group and for *Sap5* in the CF group (Figure 6). The transcript level of *Sap1* in dental biofilms from S-ECC children was higher than that from CF children (*p* = 0.001). The results from this *in vivo* model suggested that *Sap1* might play an important role in the development of S-ECC, since, in the same SAPS isoenzyme family, the sequence homology of *Sap1–Sap3* is up to 67%, and their functions and roles in the development of the disease are perhaps the same. Klinke [25] detected the expression of *Sap2* in caries lesions by immunohistochemistry, using specific monoclonal antibodies against *Sap2*, which suggested that *Sap2* may be involved in the progression of dental caries. We also found that gene expression of *Sap2*, *Sap3* and *Sap5* in the S-ECC group was higher than that in the CF group, but there was no significant difference between the S-ECC and CF groups.

Another interesting finding from our study is that the transcript level of *Sap4* in the CF group was statistically higher than that in the S-ECC group (*p <* 0.029). Some studies have confirmed that the gene expression of Saps can be regulated by environmental conditions, including temperature and pH. Furthermore, the enzyme activity of Sap is pH-dependent. For example, the optimum pH for *Sap*1–3 is pH 2.0–5.0, while for *Sap*4–6, the optimum pH is 5.0–7.0 [26]. The pH value is a critical factor for dental caries. Since lower pH may cause demineralization of enamel and dentin and lead to the development of dental caries, the virulence of strains is related to their acidogenicity and acid endurance. In our study, the gene expression of *Sap*1–3 in dental biofilm samples obtained from S-ECC children is higher than that from CF children. Moreover, the Sap activities of the S-ECC group were significantly higher than those of the CF group, measured by both the YNB–BSA agar plate method and the MTT method with BSA as the substrate (*p* < 0.05). Therefore the high level of gene expression and enzyme activity of Saps might be relalted to the lower pH environment induced by strains of *C. albicans* from S-ECC children. However, the factors which regulated the gene expression and enzyme activity of Saps still need to be further investigated.

## 3. Materials and Methods

### 3.1. Study Population

Seventy-three children aged 2.3 to 5 years old were recruited for this study and were divided into two groups: 40 children with S-ECC children and the balance CF. Written informed consent was obtained from all individuals’ families or caregivers, and the experimental procedures were approved by the Institutional Ethical Committee of the School of Stomatology, Sun Yat-sen University, Guangzhou, China. The reference number is ERC[2012]25, which was approved on 11 September 2012.

### 3.2. Sampling Procedures

Bacterial samples from pooled plaque of the S-ECC and CF children were collected as previously described [27,28]. Sterile dental probes were used to collect dental biofilm samples from all the children. For the S-ECC group, pooled samples were collected separately from caries lesions. For the CF group, pooled biofilm samples were obtained from the sound buccal surfaces and accessible proximal surfaces of molars and canines. The biofilm samples were immediately placed in sterilized tubes containing phosphate buffered saline (PBS, Boster, Wuhan, China).

### 3.3. Candida albicans Identification

For the detection of *C. albicans*, undiluted samples and 10^−1^–10^−3^ dilutions were cultured in CA medium (CHROMagar Company, Paris, France) and incubated at 37 °C under aerobic conditions for 48 h. A single typical green colony of yeast cells was obtained from the CA medium [29] and spread onto Sabouraud medium for pure culture. Twenty-six strains of *C. albicans* from S-ECC children and 14 strains from CF children were obtained.

### 3.4. Saps Enzyme Activity Determination by the Agar Plate Method

According to the method of Barros L.M. [30] all *C. albicans* isolates were tested in duplicate for verification of the enzymatic activity of Saps and phospholipases. The test medium for proteinases was BSA agar medium containing 2 g of BSA, 1.45 g of YNB (Difco Laboratories, Detroit, MI, USA), 20 g of glucose and 20 g of agar per liter of distilled water. The test medium for phospholipases consisted of SDA (Sabouraud dextrose agar) containing 57.3 g of sodium chloride, 0.55 g of calcium chloride and 100 mL of 50% sterile egg yolk (egg yolk enrichment) per liter of distilled water. Test isolates were grown on SDA for 24 h, and an inoculum of 10^7^ CFU/mL in sterile saline (absorbance 1.2 at 560 nm) was transferred to the test medium. The plates were incubated at 37 °C for 72 h. The enzymatic activity was determined by the formation of a halo around the yeast colony, and was measured in terms of the ratio of the diameter of the colony to the total diameter of the colony plus the zone of precipitation (Proteolytic activity, Pa). According to the method described by Price *et al*. [18], a *Pa* = 1.00 indicates that the test strain is negative for Saps enzyme activity. The smaller the *Pa* value, the higher the enzyme activity of Saps.

### 3.5. Saps Enzyme Activity Determination by MTT Methods

*C. albicans* strains were inoculated into Sabouraud broth plus 100 mM glucose and grown for 17 h at 37 °C in an orbital incubator (Shellab, Cornelius, OR, USA). The cells were then harvested and washed twice with 10 mL of PBS (pH 7.2) and re-suspended in the same buffer solution to yield a density of 10^7^ cells/mL (OD_540 nm_ = 0.4). A 200-μL quantity of the former candidal suspension was inoculated into 3 mL of YNB w/o ammonium sulfate in a rocker at 37 °C for 30 min. Tricarboxylic acid (TCA, Boster, Wuhan, China) was added to end the reaction. The planktonic suspension was centrifuged, and the supernatant was measured by Ultraviolet spectrophotometry (Varian, Palo Alto, CA, USA) at a wavelength of 280 nm (OD_280_). Another 200-μL quantity of the former candidal suspension was put into a 96-well plate. After the addition of 20 μL MTT, the suspension was incubated at 37 °C for 4 h. The plate was centrifuged, and the supernatant was discarded. Another 150-μL quantity of dimethyl sulfoxide (DMSO) was put into every well of a 96-well plate, and absorbance was measured by automated enzyme-linked immunosorbent assay (ELISA) at a wavelength of 490 nm (OD_490_). The Sap activity (*U* value) = OD_280_/OD_490_.

### 3.6. DNA Extraction of C. albicans

Cellular DNA of isolates was isolated by means of a whole-genome DNA extraction kit (Promega, Madison, WI, USA) according to the manufacturer’s protocols. Ultraviolet spectrophotometry (Varian, Palo Alto, CA, USA) was used to adjust DNA concentration to 10 ng/mL.

### 3.7. Genotype Determination of C. albicans by Polymerase Chain Reaction (PCR)

The primer pairs whose sequences span the site of the transposable intron in the 25S rDNA were those described by McCullough *et al*. [31]: CA-NT-L: 5'-ATAAGGGAAGTCGGCAAAATAGATCCGTAA-3' and CA-NT-R: 5'-CCTTGGCTGTGGTTTCGCTAGATAGTAGAT-3'. Amplification reactions were performed in 50 μL of distilled water containing 2.0 μL of each primer, 2.0 μL of genomic DNA (5 μg/mL) and one PCR bead (Ready-to-Go PCR beads; Amersham Pharmacia Biotech, Piscataway, NJ, USA). The PCR conditions used were as follows: denaturation by incubation for 5 min at 93 °C prior to 40 cycles of 93 °C for 30 s, 55 °C for 45 s, and 72 °C for 45 s and a final extension at 72 °C for 10 min. All reaction products were characterized by electrophoresis on 1.5% agarose gels in TBE (Tris-borate-EDTA) buffer at 70 V for 30 min and were then stained in a solution of 0.5 μg of ethidium bromide per mL.

According to the results of electrophoresis, the genotypes of *C. albicans* can be divided into 5 groups by the size of DNA amplified (450 bp for group A, 840 bp for group B, 450 and 840 bp for group C, 1080 bp for group D and 1400 bq for group E) [32,33].

### 3.8. RNA Extraction and Reverse Transcription

Total RNA was extracted and the integrity of RNA was assessed by agarose gel electrophoresis, and the purity of RNA (OD_260_/OD_280_) was measured. The reverse transcription of mRNA was performed according to the instructions for the reverse transcription kit (Promega).

### 3.9. Real-Time RT-PCR for Gene Expression of Sap1–5

Primer sets were designed to amplify *Sap1*–*Sap5* and ACT1 (actin) (Table 1). None of the primer sets amplified regions containing introns.

### 3.10. Statistical Analysis

All results were analyzed with SPSS 13.0 software (SPSS Inc., Chicago, IL, USA). The enzyme activity of Saps (*Pa* and *U* values) in the S-ECC and caries-free groups was compared by *t*-test. The *Pa* and *U* values of different genotype groups were analyzed by one-way ANOVA and the Kruskal-Wallis test. The gene expression of *Sap*1–5 (*x* ± *s*) in the S-ECC and caries-free groups was compared by the RANK test. A *p*-value < 0.05 was considered statistically significant.

## 4. Conclusions

The regulation of gene expression and enzyme activity of Sap is complicated and not clearly understood. In this *in vivo* model, we found that the enzyme activities of *Candida albicans* isolated from children with S-ECC were significantly higher than those from children in the CF group. Furthermore, the enzyme activity might be related to the genotypes of *C. albicans*, since, in the genotype A group, Saps activity in the S-ECC group was significantly different from that in the CF group. According to the results of RT-PCR, *Sap*1–5 can be detected in the biofilm from both S-ECC and CF children, but only the gene expression level of *Sap*1 in the S-ECC group was significantly higher than that in the CF group. It can thus be concluded that Saps are associated with the development of S-ECC, and that *Sap*1 may play an important role in its progression. The relationships of Saps gene expression and genotype of *C. albicans* and mechanisms of Saps gene expression regulation need to be further investigated.

## References

[1] American Academy on Pediatric Dentistry, American Academy of Pediatrics (2008). Policy on early childhood caries (ECC): Classifications, consequences, and preventive strategies. Pediatr. Dent..

[2] Berkowitz R.J. (2003). Causes, treatment and prevention of early childhood caries: A microbiologic perspective. J. Can. Dent. Assoc..

[3] Ge Y., Caufield P.W., Fisch G.S., Li Y. (2008). *Streptococcus mutans* and *Streptococcus sanguinis* colonization correlated with caries experience in children. Caries Res..

[4] Marchant S., Brailsford S.R., Twomey A.C., Roberts G.J., Beighton D. (2001). The predominant microflora of nursing caries lesions. Caries Res..

[5] Gross E.L., Leys E.J., Gasparovich S.R., Firestone N.D., Schwartzbaum J.A., Janies D.A., Asnani K., Griffen A.L. (2010). Bacterial 16S sequence analysis of severe caries in young permanent teeth. J. Clin. Microbiol..

[6] Tanner A.C., Mathney J.M., Kent R.L., Chalmers N.I., Hughes C.V., Loo C.Y., Pradhan N., Kanasi E., Hwang J., Dahlan M.A. (2011). Cultivable anaerobic microbiota of severe early childhood caries. J. Clin. Microbiol..

[7] Akdeniz B.G., Koparal E., Sen B.H., Ates M., Denizci A.A. (2002). Prevalence of *Candida albicans* in oral cavities and root canals of children. ASDC J. Dent. Child..

[8] De Carvalho F.G., Silva D.S., Hebling J., Spolidorio L.C., Spolidorio D.M. (2006). Presence of mutans *Streptococci* and *Candida* spp. in dental plaque/dentine of carious teeth and early childhood caries. Arch. Oral Biol..

[9] Rozkiewicz D., Daniluk T., Zaremba M.L., Cylwik-Rokicka D., Stokowska W., Pawinska M., Dabrowska E., Marczuk-Kolada G., Waszkiel D. (2006). Oral *Candida albicans* carriage in healthy preschool and school children. Adv. Med. Sci..

[10] Monod M., Hube B., Hess D., Sanglard D. (1998). Differential regulation of *Sap8* and *Sap9*, which encode two new members of the secreted aspartic proteinase family in *Candida albicans*. Microbiology.

[11] Monod M., Togni G., Hube B., Sanglard D. (1994). Multiplicity of genes encoding secreted aspartic proteinases in *Candida* species. Mol. Microbiol..

[12] Naglik J.R., Newport G., White T.C., Fernandes-Naglik L.L., Greenspan J.S., Greenspan D., Sweet S.P., Challacombe S.J., Agabian N. (1999). *In vivo* analysis of secreted aspartyl proteinase expression in human oral candidiasis. Infect. Immun..

[13] Schaller M., Schafer W., Korting H.C., Hube B. (1998). Differential expression of secreted aspartyl proteinases in a model of human oral candidosis and in patient samples from the oral cavity. Mol. Microbiol..

[14] Naglik J.R., Rodgers C.A., Shirlaw P.J., Dobbie J.L., Fernandes-Naglik L.L., Greenspan D., Agaban N., Challacomber S.J. (2003). Differential expression of *Candida albicans* secreted aspartyl proteinase and phospholipase B genes in humans correlates with active oral and vaginal infections. J. Infect. Dis..

[15] Schaller M., Bein M., Korting H.C., Baur S., Hamm G., Monod M., Beinhauer S., Hube B. (2003). The secreted aspartyl proteinases *Sap1* and *Sap2* cause tissue damage in an *in vitro* model of vaginal candidiasis based on reconstituted human vaginal epithelium. Infect. Immun..

[16] Staib P., Kretschmar M., Nichterlein T., Hof H., Morschhauser J. (2000). Differential activation of a *Candida albicans* virulence gene family during infection. Proc. Natl. Acad. Sci. USA.

[17] Kretschmar M., Felk A., Staib P., Schaller M., Hess D., Callapina M., Morschhauser J., Schafer W., Korting H.C., Hof H. (2002). Individual acid aspartic proteinases (Saps) 1–6 of *Candida albicans* are not essential for invasion and colonization of the gastrointestinal tract in mice. Microb. Pathog..

[18] Price M.F., Wilkinson I.D., Gentry L.O. (1982). Plate method for detection of phospholipase activity in *Candida albicans*. Sabouraudia.

[19] Yang X.Q., Zhang Q., Lu L.Y., Yang R., Liu Y., Zou J. (2012). Genotypic distribution of *Candida albicans* in dental biofilm of chinese children associated with severe early childhood caries. Arch. Oral Biol..

[20] Al-Karaawi Z.M., Manfredi M., Waugh A.C., McCullough M.J., Jorge J., Scully C., Porter S.R. (2002). Molecular characterization of *Candida* spp. isolated from the oral cavities of patients from diverse clinical settings. Oral Microbiol. Immunol..

[21] Hube B., Monod M., Schofield D.A., Brown A.J., Gow N.A. (1994). Expression of seven members of the gene family encoding secretory aspartyl proteinases in *Candida albicans*. Mol. Microbiol..

[22] White T.C., Agabian N. (1995). *Candida albicans* secreted aspartyl proteinases: Isoenzyme pattern is determined by cell type, and levels are determined by environmental factors. J. Bacteriol..

[23] Ripeau J.S., Fiorillo M., Aumont F., Belhumeur P., de Repentigny L. (2002). Evidence for differential expression of *Candida albicans* virulence genes during oral infection in intact and human immunodeficiency virus Type 1-Transgenic mice. J. Infect. Dis..

[24] Schaller M., Schackert C., Korting H.C., Januschke E., Hube B. (2000). Invasion of *Candida albicans* correlates with expression of secreted aspartic proteinases during experimental infection of human epidermis. J. Investig. Dermatol..

[25] Klinke H.T., Pönisch R., Kriegel T.M., Klimm H.W. (2007). Immunohistochemical detection of the collagenolytic *Candia albicans Sap2* proteinase in caries lesions. Caries Res..

[26] Borg-von Z.M., Beggah S., Boggian K., Sanglard D., Monod M. (1998). The expression of the secreted aspartic proteinases *Sap4* to *Sap6* from *Candida albicans* in murine macrophages. Mol. Microb..

[27] Caufield P.W., Saxena D., Fitch D., Li Y. (2007). Population structure of lasmid-containing strains of *Streptococcus mutans*, a member of the human indigenous biota. J. Bacteriol..

[28] Li Y., Ge Y., Saxena D., Caufield P.W. (2007). Genetic profiling of the oral microbiota associated with severe early childhood caries. J. Clin. Microbiol..

[29] Odds F.C., Bernaerts R. (1994). CHROMagar *Candida*, a new differential isolation medium for presumptive identification of clinically important candida species. J. Clin. Microbiol..

[30] Barros L.M., Boriollo M.F., Alves A.C., Klein M.I., Gonçalves R.B., Hofling J.F. (2008). Genetic diversity and exoenzyme activities of *Candida albicans* and *Candida dubliniensis* isolated from the oral cavity of brazilian periodontal patients. Arch. Oral Biol..

[31] McCullough M.J., Clemons K.V., Stevens D.A. (1999). Molecular and phenotypic characterization of genotypic *Candida albicans* subgroups and comparison with *Candida dubliniensis* and *Candida stellatoidea*. J. Clin. Microbiol..

[32] She X.D., Wang X.J., Fu M.H., Shen Y.N., Liu W.D. (2008). Genotype comparisons of strains of *Candida albicans* from patients with cutaneous candidiasis and vaginal candidiasis. Chin. Med. J..

[33] Tamura M., Watanabe K., Mikami Y., Yazawa K., Nishimura K. (2001). Molecular characterization of new clinical isolates of *Candida albicans* and *C. dubliniensis* in Japan: Analysis reveals a new genotype of *C. albicans* with Group I Intron. J. Clin. Microbiol..

